# Effects of modern physical training on physical performance and training-related injuries in the Chinese people’s armed police force: a systematic review and meta-analysis

**DOI:** 10.3389/fphys.2026.1953076

**Published:** 2026-09-14

**Authors:** Yilin Wang, Jiehao Cai, Xiantao Huang

**Affiliations:** 1 School of Physical Education, Wuhan University of Science and Technology, Wuhan, China; 2 School of Physical Education, Shenzhen University, Shenzhen, China

**Keywords:** Chinese people’s armed police force, meta-analysis, modern physical training, physical fitness, systematic review, training-related injuries

## Abstract

**Background:**

The Chinese People’s Armed Police Force (PAP) undertakes high-intensity counter-terrorism, stability-maintenance and security tasks requiring exceptional physical fitness. However, conventional military physical training is monotonous in design, and training-related injury rates remain high (3,25). Modern physical training paradigms—Comprehensive Physical Training (CPT) and Preventive Training (PT)—have been gradually introduced into the PAP system, yet their combined effects have not been systematically quantified.

**Objective:**

To evaluate the effects of modern physical training (CPT and PT) compared with conventional military training on physical performance (strength, endurance, speed, agility), movement quality (as measured by the Functional Movement Screen [FMS]), body composition (BMI) and training-related injury rates in PAP personnel.

**Methods:**

This review followed PRISMA 2020 guidelines and the PROSPERO protocol (CRD420261448648). We searched PubMed, Embase, CENTRAL, SCIE, Scopus, PsycINFO, Google Scholar, CNKI, Wanfang and Weipu databases without date or language restrictions. We included randomized controlled trials (RCTs) and high-quality non-randomized studies comparing modern physical training (CPT/PT) with conventional military training in PAP personnel, reporting at least one physical fitness outcome or training-related injury rate. Two reviewers independently performed screening, data extraction, and risk of bias assessment (RoB 2.0 for RCTs; ROBINS-I for non-randomized studies). Continuous outcomes were expressed as standardized mean differences (SMD) and dichotomous outcomes as risk ratios (RR), both with 95% confidence intervals, using Hartung-Knapp random-effects models. Prespecified subgroup analyses were performed by population (recruits vs. veterans/cadets) and intervention duration (<12 vs. ≥12 weeks). Evidence quality was assessed using GRADE.

**Results:**

After screening 1,739 records, 20 studies (n = 4,158 PAP personnel) were included in the meta-analysis. Modern physical training significantly outperformed conventional training in pull-ups (19 studies, SMD = 0.68 [0.50, 0.85], I^2^ = 76.5%), 3000m run (15 studies, SMD = −0.87 [−1.40, −0.33]; however, after excluding the extreme outlier Yan (2024), the effect remained significant (SMD = −0.52, 95% CI [−0.68, −0.36]) with substantially reduced heterogeneity I^2^ =50.6%), 100m sprint (10 studies, SMD = −0.42 [−0.65, −0.19]), 30m×2 zigzag run (6 studies, SMD = −0.82 [−1.16, −0.48]), 2-min sit-ups (9 studies, SMD = 0.57 [0.24, 0.89]), parallel bar dips (7 studies, SMD = 0.58 [0.16, 0.99], I^2^ = 74.9%), FMS total score (4 studies, SMD = 1.92 [0.68, 3.17]) and BMI (5 studies, SMD = 0.19 [0.03, 0.36], I^2^ = 0%). Training-related injury rate (4 studies, RR = 0.63 [0.46, 0.87]) was reduced by 37%. FMS showed the largest effect in <12-week FMS-specific training (SMD = 2.45), significantly higher than the ≥12-week comprehensive programs (SMD = 0.90, subgroup difference p < 0.001).

**Conclusion:**

Modern physical training (CPT and PT) significantly enhances physical performance across multiple dimensions—including upper body strength, upper body pushing power, core endurance, aerobic capacity, speed, agility and functional movement—and favorably modifies body composition, while reducing the risk of training-related injuries by approximately 37% (RR = 0.63). FMS screening and targeted corrective training is recommended during recruit training (<12 weeks), while periodized comprehensive physical training programs are recommended for ongoing training (≥12 weeks). These findings provide evidence-based guidance for optimizing training protocols and enhancing combat readiness in PAP units.

**Systematic review registration:**

https://www.crd.york.ac.uk/PROSPERO/view/, identifier CRD420261448648.

## 1 Introduction

### 1.1 Specificity and challenges of physical training in the PAP

The Chinese People’s Armed Police Force (PAP) is an independent internal security force separate from the People’s Liberation Army, tasked with stability maintenance, counter-terrorism, emergency response, and major event security. These operational demands impose stringent physical fitness requirements on its personnel (Zhang and Chen, 2005; State Council of the PRC and Central Military Commission, 2009).

However, conventional PAP physical training has historically emphasized long-distance running, push-ups, pull-ups and drill routines. This approach not only constrains fitness improvements but is also associated with persistently high training-related injury rates. Injury rates of 20–25% have been reported during basic combat training in the US military (Knapik et al., 2001), and comparable rates have been documented among PAP recruits during initial training (Hao, 2005; Zhang et al., 2011). Overuse injuries such as stress fractures, tendinopathy and muscle strains predominate (Knapik et al., 2001). Designing training programs that simultaneously enhance physical performance and reduce injury risk has become a central challenge in PAP training reform (Duan, 2014; Sulsky et al., 2018).

### 1.2 Advances in modern physical training

In recent years, several physical training paradigms have been progressively introduced into military physical training alongside conventional regimens. Based on the existing literature, these paradigms can be classified into two broad categories. The first is Comprehensive Physical Training (CPT), which aims to develop multiple fitness components within a single program, and includes: (i) the Extreme Conditioning Program (ECP) (Yan, 2024), a high-intensity, constantly-varied training format combining weightlifting, gymnastics and metabolic conditioning performed at high work rates with minimal rest; (ii) periodized training (Li, 2022), in which training volume, intensity and content are systematically varied across defined phases (e.g., accumulation, intensification and tapering) to optimize adaptation and reduce stagnation; (iii) lactate-threshold training (Cao, 2021), an aerobic endurance method in which exercise intensity is prescribed relative to the individual lactate threshold—typically monitored by heart rate or running velocity—to improve submaximal endurance; and (iv) circuit strength training (Guo, 2020), which sequences multiple resistance exercises with short inter-set rest intervals to develop both muscular strength and endurance. The second category is Preventive Training (PT), which targets injury risk reduction and movement quality, and includes: (i) FMS-guided corrective exercise (Wu et al., 2021; Zhang, 2022), in which a Functional Movement Screen (FMS) identifies movement-pattern deficiencies that are subsequently addressed with targeted corrective exercises; (ii) functional training (Li, 2021; Wang, 2021), a task-oriented approach emphasizing multi-joint, multi-planar movements and proprioceptive integration rather than isolated single-joint actions; and (iii) comprehensive medical interventions (Hao, 2005; Zhang et al., 2011), combining medical supervision, health education and training-load monitoring throughout the training cycle. It is important to acknowledge that conventional PAP training—long-distance running, push-ups, pull-ups and drill routines—provides a fundamental and structured physical foundation, and its components remain the building blocks of any conditioning program. Our review therefore does not assert that modern paradigms are inherently superior in every context; rather, it evaluates whether specific additions, re-organizations or targeted corrections of training content yield measurable improvements in physical performance and injury outcomes. International evidence, such as studies on high-intensity functional training (HIFT), has similarly suggested advantages of these approaches in military populations (Helén et al., 2023). While individual studies indicate that such paradigms may outperform conventional training, they are limited by small sample sizes, heterogeneous designs and inconsistent outcome measures, and their overall effects have not been quantitatively synthesized with high-quality evidence. International military readiness frameworks have emphasized the need for evidence-based, periodized training programs to optimize soldier performance (Nindl et al., 2018). Three similar PROSPERO registrations (CRD42025636689, CRD420261442021, CRD420261415027) were identified, but none focused specifically on the PAP population or examined both physical performance and injury outcomes simultaneously.

### 1.3 Study rationale and objectives

To date, no systematic review and meta-analysis has specifically targeted the Chinese PAP and systematically quantified the combined effects of modern physical training (CPT and PT) using the PICOS framework. This study therefore aimed to address the following questions: (1) Compared with conventional military training, can modern physical training significantly improve physical performance and reduce training-related injury rates in PAP personnel? (2) Do these effects differ by population (recruits vs. veterans/cadets) or intervention duration (<12 vs. ≥12 weeks)? We hypothesized that modern physical training would be superior to conventional training across all physical fitness domains (strength, endurance, speed, agility, FMS) and would significantly reduce the risk of training-related injuries.

## 2 Methods

### 2.1 Protocol and registration

This systematic review adheres to the PRISMA 2020 guidelines (Page et al., 2021). The protocol was prospectively registered on PROSPERO on July 12, 2026 (registration: CRD420261448648, version 2.0). Version 2 revised the pooling method from mean difference (MD) to standardized mean difference (SMD) and removed the subgroup analysis by intervention type.

### 2.2 Search strategy

We systematically searched eight databases: PubMed, Embase, CENTRAL, SCIE, Scopus, PsycINFO, Google Scholar, and CNKI, and supplemented with Wanfang, VIP and the CNKI dissertation databases to cover grey literature. Reference lists of included studies were hand-searched (backward citation), and forward citation tracking was also performed. The search strategy was built on the PICOS framework using both MeSH terms and free-text keywords, including “Chinese People’s Armed Police Force”, “PAP”, “武警”, “physical training”, “military personnel” and “randomized controlled trial” (full strategy available in the PROSPERO protocol). No date or language restrictions were applied.

### 2.3 Eligibility criteria

The inclusion criteria followed the PICOS framework: (P) PAP officers and soldiers (recruits, veterans, cadets), aged 17–29 years; (I) modern physical training interventions, including CPT (ECP, periodized training, lactate-threshold training, pattern training) and PT (functional training, FMS-guided corrective exercise, core stability training, comprehensive medical intervention); (C) conventional military physical training as the comparator; (O) at least one physical fitness outcome (pull-ups, 2-min sit-ups, 3000m run, 100m sprint, 30m×2 zigzag run, FMS total score, BMI, or parallel bar dips) or training-related injury rate (reported as event counts with sample sizes); (S) RCTs and non-randomized controlled trials (including cluster-RCTs and stratified RCTs). Exclusion criteria: single-arm studies without a control group; non-PAP populations; reviews, case reports, conference abstracts, and duplicate publications.

In this review, recruits were defined as personnel undergoing initial basic training; veterans were defined as personnel who had completed initial basic training and were serving in regular units; and cadets were defined as officer candidates enrolled in military academies.

### 2.4 Study selection and data extraction

Two reviewers (Y.W. and J.C.) independently screened titles and abstracts, then assessed full texts of potentially eligible studies. Disagreements were resolved through discussion or by a third reviewer. Data extracted included: first author and year, sample sizes (EG, experimental group; CG, control group), intervention type (CPT or PT), population (recruits, veterans, or cadets), intervention duration (weeks and <12/≥12 weeks classification), post-intervention outcome data (means and SDs for continuous variables; event counts and sample sizes for dichotomous variables), study design, and P values. Authors were contacted for missing data when necessary.

For each included study, we additionally extracted the characteristics of the conventional (control) training program, including program content, frequency, volume, intensity, periodization/progression, and duration, where reported by the original studies (**Supplementary Table 4** in the Supplementary Materials).

### 2.5 Risk of bias and quality assessment

Two reviewers independently assessed risk of bias. RCTs were evaluated using the Cochrane Risk of Bias 2.0 tool (RoB 2.0) across five domains: randomization process, deviations from intended interventions, missing outcome data, measurement of the outcome, and selection of the reported result. Non-randomized studies were assessed using the ROBINS-I tool across seven domains: confounding, participant selection, intervention classification, deviations, missing data, outcome measurement, and selective reporting. Overall evidence quality was evaluated using the GRADE approach, rating each outcome as high, moderate, low, or very low certainty based on risk of bias, inconsistency, indirectness, imprecision and publication bias.

### 2.6 Statistical analysis

All analyses were conducted in R software (version 4.5.3) using the meta and metafor packages. Continuous outcomes were pooled using the standardized mean difference (SMD, Hedges’ g correction) to accommodate different measurement scales; dichotomous outcomes were pooled using the risk ratio (RR). All effect estimates are presented with 95% confidence intervals. Given anticipated heterogeneity in training programs, populations, and testing protocols, we used the Hartung-Knapp-Sidak random-effects model (restricted maximum likelihood estimation) to control Type I error inflation in meta-analyses with few studies. Heterogeneity was assessed using the I^2^ statistic (>50% indicating substantial heterogeneity), τ^2^ estimate, and Cochran’s Q test (p < 0.10 considered significant). Effect sizes were interpreted using Cohen’s conventions: 0.2 = small, 0.5 = medium, 0.8 = large.

Two subgroup analyses were predefined: (1) by population (recruits vs. veterans/cadets); veterans and cadets were pooled into a single subgroup because both groups had completed initial recruit training and possessed established physical fitness baselines, whereas recruits were undergoing their first systematic conditioning cycle. This distinction captures the differential training responsiveness of deconditioned versus conditioned populations; and (2) by intervention duration (<12 vs. ≥12 weeks). Sensitivity analyses included: (1) leave-one-out analysis; (2) excluding non-randomized studies to assess the robustness of findings in RCTs only; and (3) excluding extreme outliers (e.g., Yan, 2024 for the 3000m run). Publication bias was assessed by visual inspection of funnel plots and Egger’s regression test when ≥10 studies were available (p < 0.05 indicating significant asymmetry). The trim-and-fill method was applied to estimate adjusted effect sizes when asymmetry was detected. Outliers were defined as studies whose 95% CIs did not overlap with the pooled estimate.

## 3 Results

### 3.1 Study selection

The initial search yielded 1,739 records. After removing 535 duplicates, 1,204 records were screened for title and abstract, with 1,128 records excluded. Seventy-six full-text articles were assessed for eligibility, of which 20 studies (n = 4,158 PAP personnel) ultimately met the inclusion criteria. Exclusion reasons included: non-PAP population (38 articles), no adequate control group (12 articles), and insufficient data (6 articles). The PRISMA flow diagram is presented in **Figure 1**.

**Figure 1:**
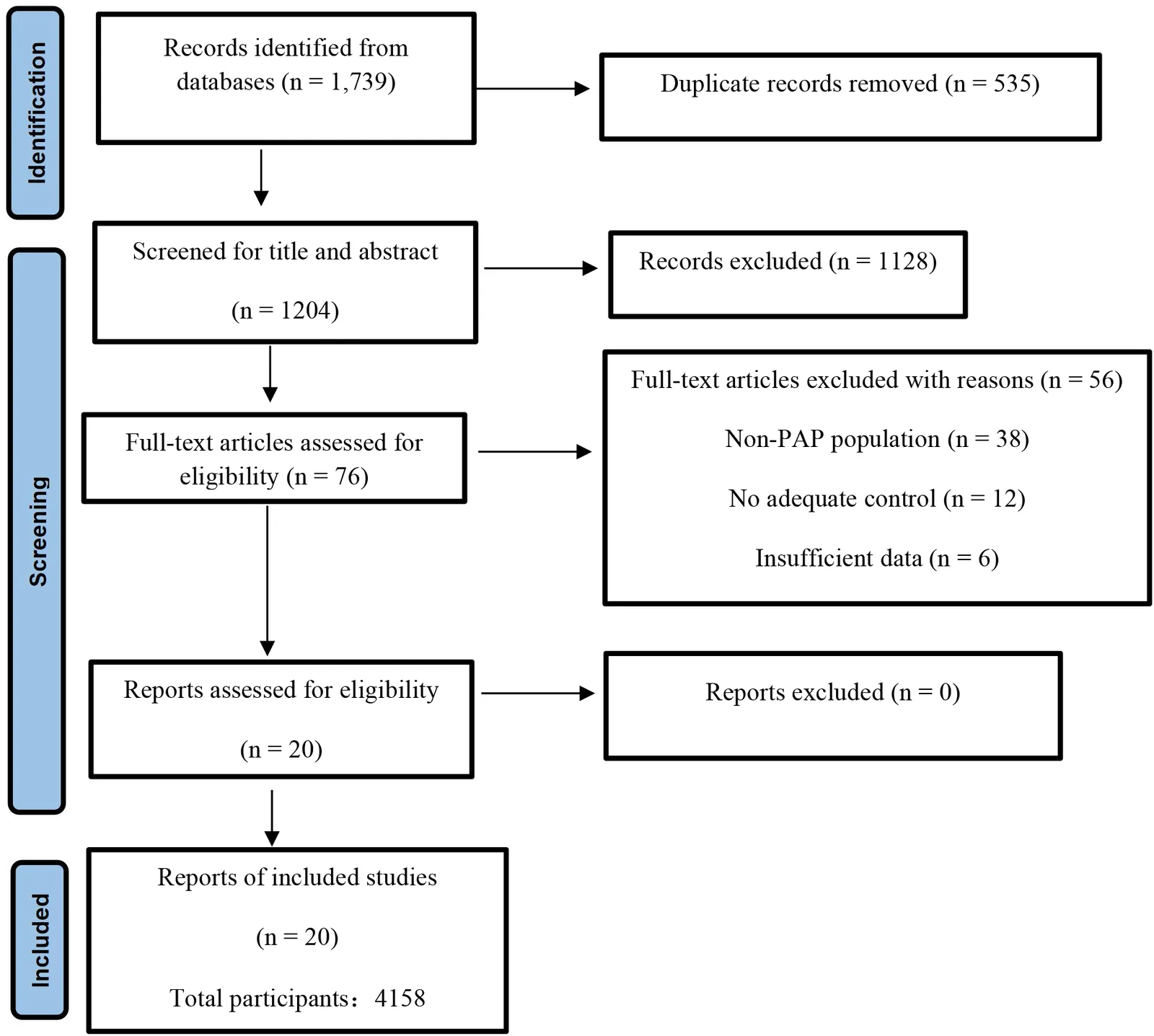
PRISMA 2020 flow diagram of the study selection process.

### 3.2 Study characteristics

The 20 included studies were published between 2005 and 2024, with sample sizes ranging from 16 to 943 participants. The designs comprised 16 RCTs, 1 stratified RCT (Hao, 2005), 1 cluster RCT (Liu, 2017), and 2 non-randomized controlled trials (Zhang et al., 2011; Wu et al., 2021). Thirteen studies employed CPT interventions (Zhang, 2005; Zhang et al., 2005a; Zhang et al., 2005b; Duan, 2014; Zhang et al., 2017; Chen, 2020; Guo, 2020; Hu, 2021; Li, 2021; Zhou, 2021; Chen, 2022; Li, 2022; Yan, 2024) and 7 used PT interventions (Hao, 2005; Zhang et al., 2011; Liu, 2017; Cao, 2021; Wang, 2021; Wu et al., 2021; Zhang, 2022). Eleven studies targeted recruits, 7 targeted veterans, and 2 targeted cadets. Intervention durations were <12 weeks in 11 studies and ≥12 weeks in 9 studies. Detailed characteristics are shown in **Table 1**.

**Table 1:** Characteristics of the included studies.

Study	Population	Design	Duration (weeks)	Sample size (n, EG/CG)	Training type	Key outcomes
Yan, 2024	Recruits	RCT	≥12	200 (100/100)	CPT	BMI, FMS, 3000m, pull-ups, parallel bar dips, sit-ups
Li, 2022	Veterans	RCT	≥12	120 (60/60)	CPT	100m, 3000m, pull-ups, zigzag run
Chen, 2020	Recruits	RCT	<12	44 (22/22)	CPT	100m, 3000m, parallel bar dips, pull-ups, sit-ups
Zhou, 2021	Recruits	RCT	≥12	61 (31/30)	CPT	3000m, BMI, pull-ups, sit-ups, zigzag run
Guo, 2020	Recruits	RCT	<12	140 (70/70)	CPT	100m, 3000m, parallel bar dips, pull-ups, sit-ups, zigzag run
Wu et al., 2021	Recruits	NRS	<12	316 (158/158)	PT	100m, 3000m, parallel bar dips, pull-ups, sit-ups, training injury
Zhang et al., 2005a	Recruits	RCT	<12	400 (200/200)	CPT	100m, 3000m, pull-ups
Zhang et al., 2005c	Veterans	RCT	<12	210 (120/90)	CPT	100m, 3000m, pull-ups
Zhang et al., 2005b	Cadets	RCT	<12	380 (200/180)	CPT	100m, 3000m, pull-ups
Zhang, 2022	Recruits	RCT	<12	60 (30/30)	PT	3000m, FMS, parallel bar dips, pull-ups, training injury
Wang, 2021	Recruits	RCT	≥12	150 (75/75)	PT	BMI, FMS, 3000m, pull-ups, sit-ups, zigzag run
Li et al., 2021	Veterans	RCT	<12	50 (26/24)	CPT	3000m, pull-ups, sit-ups, zigzag run
Zhang et al., 2017	Veterans	RCT	<12	40 (20/20)	CPT	pull-ups
Hao, 2005	Recruits	Strat. RCT	≥12	835 (420/415)	PT	100m, 3000m, pull-ups, training injury
Hu, 2021	Veterans	RCT	<12	60 (30/30)	CPT	100m, pull-ups
Duan, 2014	Recruits	RCT	≥12	56 (28/28)	CPT	100m, 3000m, parallel bar dips, pull-ups, sit-ups
Liu, 2017	Veterans	Cluster RCT	<12	41 (20/21)	PT	100m, parallel bar dips, sit-ups
Chen, 2022	Recruits	RCT	≥12	150 (75/75)	CPT	BMI, FMS, 3000m, pull-ups, sit-ups, zigzag run
Cao, 2021	Recruits	RCT	≥12	16 (8/8)	CPT	BMI, 3000m, pull-ups, sit-ups
Zhang et al., 2011	Veterans	NRS	<12	943 (429/514)	PT	training injury

CPT, Comprehensive Physical Training; PT, Preventive Training; RCT, Randomized Controlled Trial; NRS, Non-Randomized Study; EG, Experimental Group; CG, Control Group.

Studies Zhang et al. (2005a), (2005b), and (2005c) are three independent cohorts from the same 8-week protocol. Duration ≥12 weeks includes 3-month (Hao, 2005) and 6-month (Zhang et al., 2011) programs. Detailed descriptions of interventions and controls are provided in **Supplementary Table 1**.

### 3.3 Risk of bias and evidence quality

RoB 2.0 assessment of 18 RCTs showed that approximately 60% of studies were rated as “low risk” or “some concerns,” while approximately 40% were rated as “high risk of bias” in at least one domain, primarily deviations from intended interventions (D2) and missing outcome data (D3), reflecting the inherent challenges of blinding in exercise training studies and participant attrition during recruit training. The two non-randomized studies were assessed as “moderate risk” on ROBINS-I, with primary concerns in the confounding domain (Zhang et al., 2011; Wu et al., 2021).

GRADE evidence quality was rated as “moderate” for pull-ups and training-related injury rate; parallel bar dips was rated “low” (downgraded one level for risk of bias or heterogeneity), “low” for FMS total score and 3000m run (downgraded for high heterogeneity), and “very low” to “low” for the remaining outcomes. The risk of bias summary is shown in **Figure 2**.

**Figure 2:**
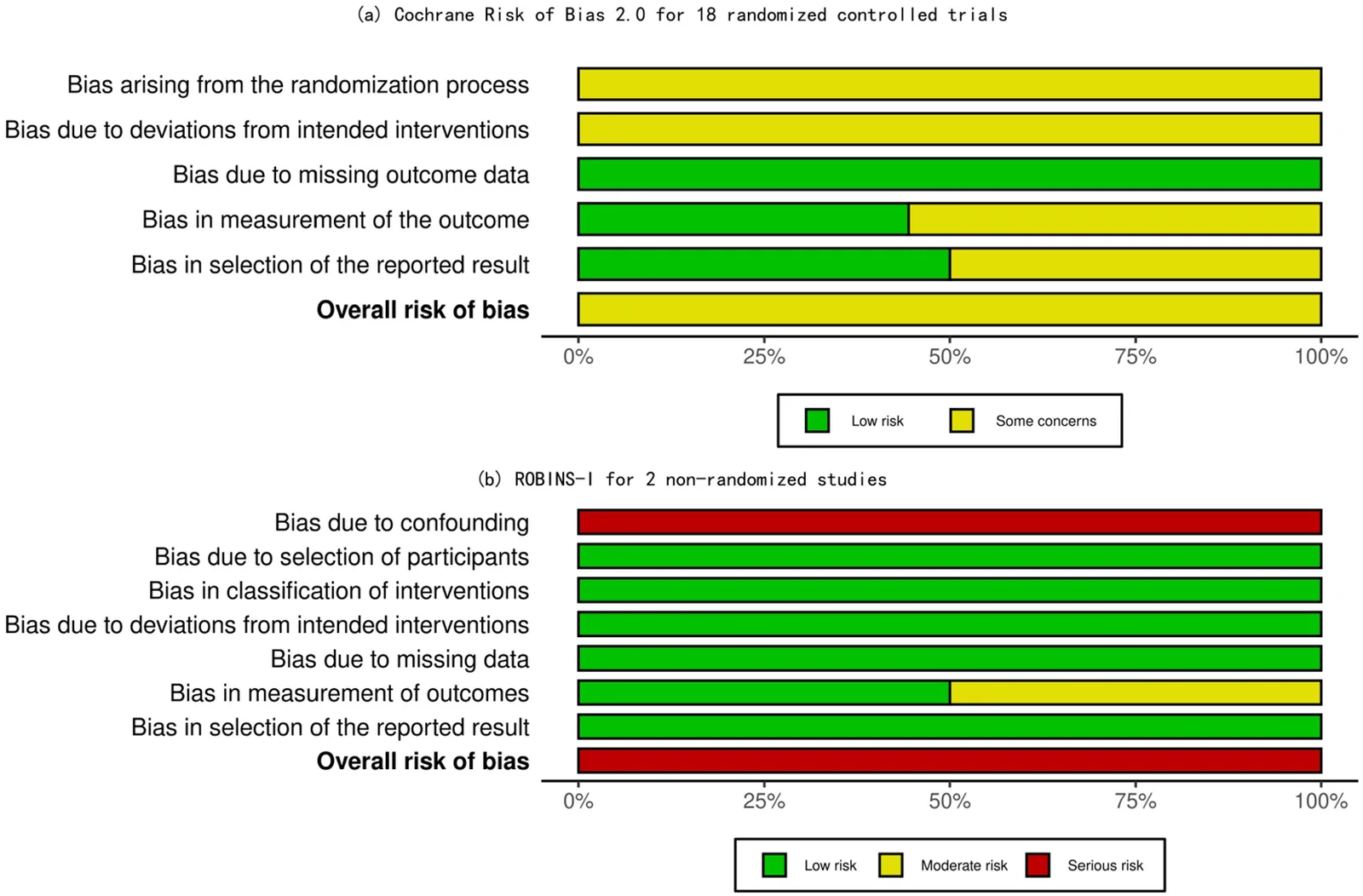
Summary of risk of bias assessment (RoB 2.0 and ROBINS-I).

### 3.4 Meta-analysis of physical performance outcomes

#### 3.4.1 Pull-ups (upper body pulling strength)

Nineteen studies (n = 3,233) reported pull-up performance. The random-effects model showed that modern physical training was significantly superior to conventional training (SMD = 0.68, 95% CI [0.50, 0.85], p < 0.0001, I^2^ = 76.5%). Subgroup analysis by population: recruits SMD = 0.64 [0.43, 0.85]; veterans SMD = 0.81 [0.41, 1.21]; cadets SMD = 0.46 [0.25, 0.66]. Subgroup analysis by duration: <12 weeks SMD = 0.71 [0.47, 0.94]; ≥12 weeks SMD = 0.65 [0.40, 0.90]. Leave-one-out sensitivity analysis confirmed stability (range 0.63–0.72). Egger’s test was non-significant (p = 0.182). See **Figure 3**.

**Figure 3:**
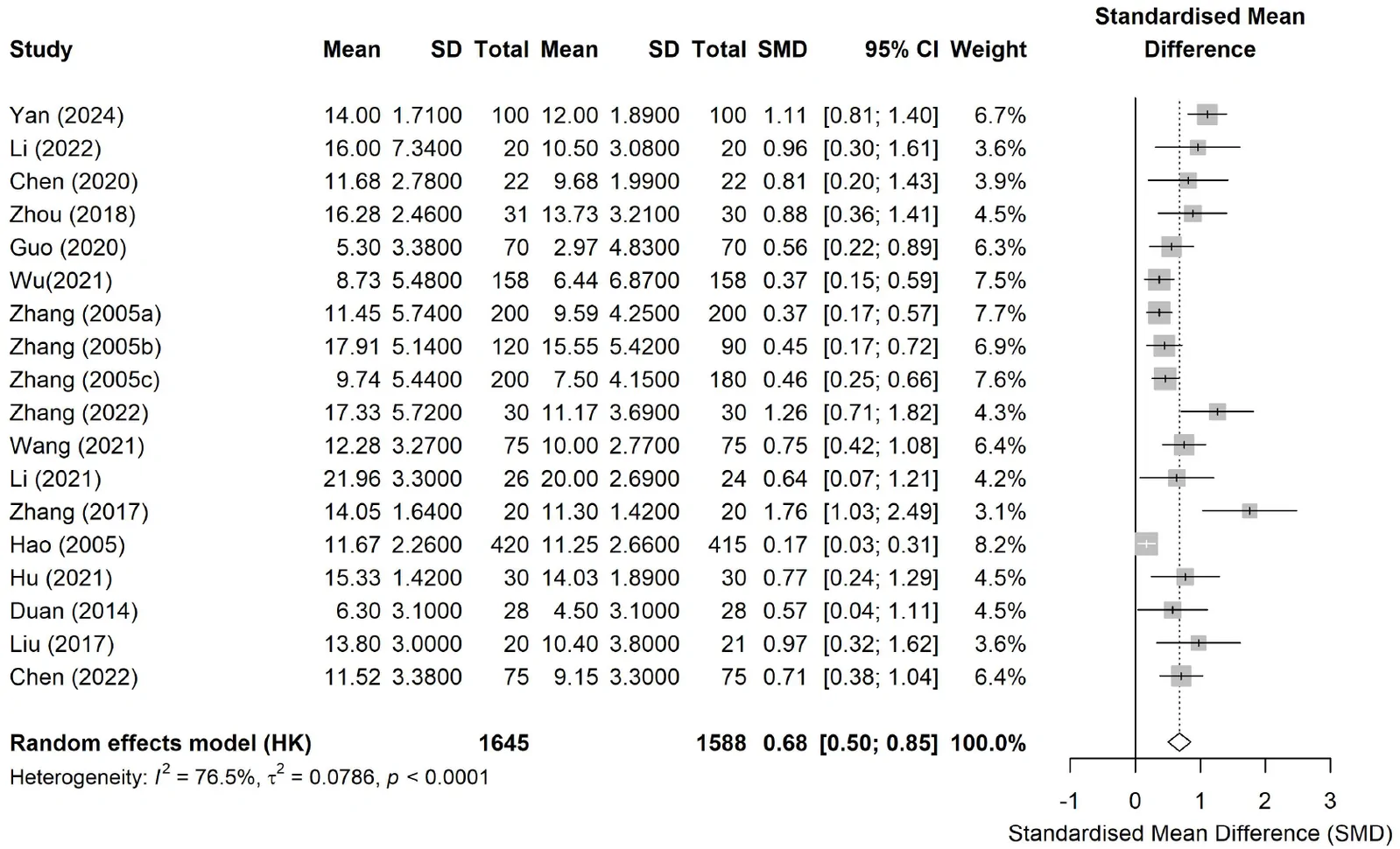
Forest plot of pull-ups.

#### 3.4.2 Parallel bar dips (upper body pushing strength)

Seven studies (n = 725) reported parallel bar dips. The pooled effect showed a moderate but significant advantage for modern training (SMD = 0.58 [0.16, 0.99], I^2^ = 74.9%).

#### 3.4.3 2-minute sit-ups (core endurance)

Nine studies (n = 1,167) reported 2-minute sit-ups. The random-effects model yielded a moderate effect (SMD = 0.57 [0.24, 0.89], p < 0.0001, I^2^ = 81.1%).

#### 3.4.4 3000m run (aerobic endurance)

Fifteen studies (n = 2,273) reported 3000m run times. The pooled effect favored modern training (SMD = −0.87 [−1.40, −0.33], p < 0.0001), but heterogeneity was the highest among all outcomes (I^2^ = 94.0%). Given the extreme effect of Yan (2024) (Yan, 2024) (SMD = −4.12), a sensitivity analysis excluding this study was performed, yielding SMD = −0.52 [−0.68, −0.36] with substantially reduced heterogeneity (I^2^ = 50.6%). Subgroup effects were consistent across recruits (SMD = −0.79), veterans (SMD = −1.00) and cadets (SMD = −0.58). Egger’s test was non-significant (p = 0.10).

#### 3.4.5 100m sprint (speed)

Ten studies (n = 2,206) reported 100m sprint times. Modern training significantly improved sprint performance (SMD = −0.42 [−0.65, −0.19], p = 0.0035, I^2^ = 63.3%). The effect was larger in the ≥12-week subgroup (SMD = −0.61) than in the <12-week subgroup (SMD = −0.35). Egger’s test was non-significant.

#### 3.4.6 30m × 2 zigzag run (agility)

Six studies (n = 591) reported zigzag run performance. The pooled effect favored modern training (SMD = −0.82 [−1.16, −0.48], I^2^ = 45.6%), indicating a large effect on agility.

#### 3.4.7 FMS total score (functional movement)

Four studies (n = 560) reported Functional Movement Screen (FMS) total scores, which integrate seven movement-pattern sub-scores (deep squat, hurdle step, inline lunge, shoulder mobility, active straight-leg raise, trunk stability push-up, and rotational stability). The pooled effect was the largest among all outcomes (SMD = 1.92 [0.68, 3.17], p < 0.0001), but heterogeneity was very high (I^2^ = 93.2%). This heterogeneity was entirely explained by differences in training approach: Yan (2024) (Yan, 2024) used comprehensive ECP (SMD = 0.90), whereas the other three studies (Li, 2021; Wang, 2021; Zhang, 2022) employed FMS-specific corrective programs (SMD ≈ 2.36). After removing Yan (2024), the pooled SMD increased to 2.36 [2.07, 2.65] with I^2^ = 0%. Duration subgroup analysis showed that <12-week FMS-specific training yielded a much larger effect (SMD = 2.45 [2.13, 2.78], I^2^ = 0%) than ≥12-week comprehensive programs (SMD = 0.90 [0.61, 1.19]), with a significant subgroup difference (p < 0.001), highlighting the “short-cycle, specialty-specific” nature of FMS improvement.

#### 3.4.8 BMI

Five studies (n = 577) reported BMI. At baseline, mean BMI values ranged from 21.2 to 23.8 kg/m^2^ across groups, with no significant between-group differences within any study. After the interventions, BMI decreased in both arms in all four studies that reported continuous data, but the reduction was consistently equal to or larger in the conventional training group (e.g., Yan, 2024: −1.77 vs. −0.93 kg/m^2^; Cao, 2021: −0.86 vs. −0.36 kg/m^2^; Zhou (2021): −1.17 vs. −1.15 kg/m^2^), whereas the fifth study (Wang, 2021) reported only pass-rate data (100% vs. 89% pass). The pooled effect was statistically significant but small (SMD = 0.19 [0.03, 0.36], p = 0.03, I^2^ = 0%), with limited clinical relevance. The positive direction indicates a slightly higher BMI in the modern-training group, which—in the absence of body-composition data—may reflect greater preservation of lean mass alongside fat loss in resistance- and function-oriented training, rather than a lack of weight control; given that baseline BMI values were already within the normal range (18.5–23.9 kg/m^2^) in the majority of participants, further reductions in BMI would not necessarily confer additional health benefits. This warrants cautious interpretation.

### 3.5 Training-related injury rate

Four studies (n = 2,154) reported training-related injury events. Modern physical training significantly reduced injury risk (RR = 0.63 [0.46, 0.87], p = 0.005, I^2^ = 9.9%), representing a 37% risk reduction with very low heterogeneity. Subgroup analysis by intervention type confirmed that PT interventions alone also significantly reduced injury rates (RR = 0.65 [0.46, 0.91]). Restricting to RCTs only yielded RR = 0.69 [0.15, 3.12], which, although statistically non-significant due to the limited number of included RCTs (k=2), showed a consistent protective trend with the primary analysis, and the test for subgroup differences was not significant (p = 0.41). Pooled results for all nine outcomes are summarized in **Table 2**, and the subgroup dot plot is presented in **Figure 4**.

**Table 2:** Summary of meta-analysis results for all nine outcomes.

Outcome	Effect measure	Studies (k)	Sample size (n)	Pooled effect [95% CI]	Heterogeneity I^2^ (p)	Test for overall effect Z (p)
Pull-ups	SMD	19	3,233	0.68 [0.50,0.85]	76.5% (p<0.001)	Z=7.48 (p<0.0001)
Parallel bar dips	SMD	7	725	0.58 [0.16, 0.99]	74.9% (p<0.0001)	Z=2.69 (p=0.007)
2-min sit-ups	SMD	9	1,167	0.57 [0.24,0.89]	81.1% (p<0.0001)	Z=3.30 (p=0.001)
3000m run	SMD	15	2,273	−0.87 [−1.40,−0.33]	94.0% (p<0.0001)	Z=3.14 (p=0.002)
100m sprint	SMD	10	2,206	−0.42 [−0.65,−0.19]	63.3% (p=0.0035)	Z=3.52 (p<0.001)
30m×2 zigzag run	SMD	6	591	−0.82 [−1.16,−0.48]	45.6% (p=0.102)	Z=4.72 (p<0.0001)
FMS total score	SMD	4	560	1.92 [0.68,3.17]	93.2% (p<0.0001)	Z=3.04 (p=0.002)
BMI	SMD	5	577	0.19 [0.03,0.36]	0% (p=0.744)	Z=2.28 (p=0.023)
Training injury rate	RR	4	2,154	0.63 [0.46,0.87]	9.9% (p=0.344)	Z=2.84 (p=0.019)
Injury rate (RCTs only)	RR	3	1,211	0.69 [0.15, 3.12]	0% (p = 0.41)	Z=0.46 (p=0.65)

**Figure 4:**
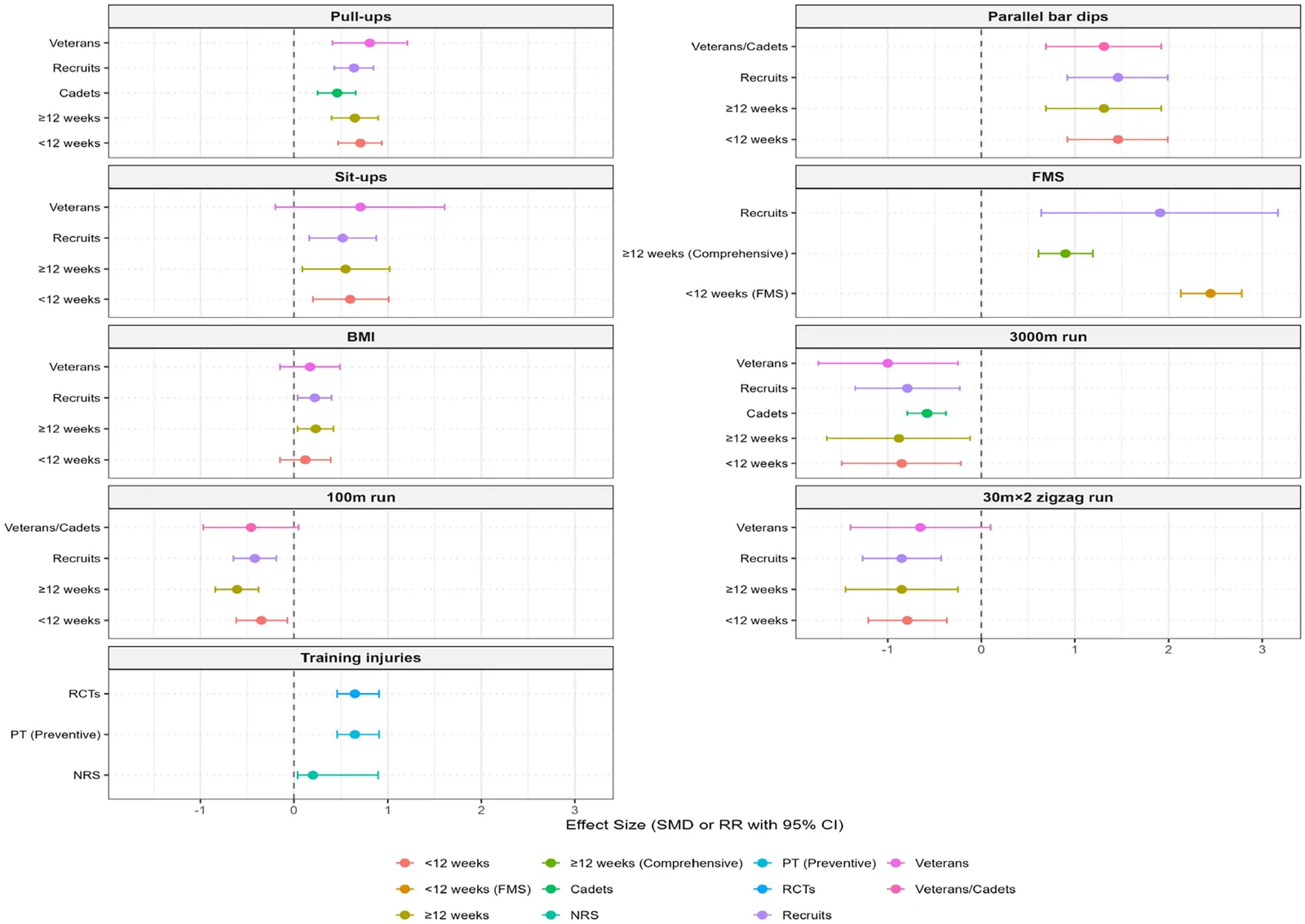
Subgroup analysis dot plot.

### 3.6 Sensitivity analysis

Sensitivity analysis confirmed that the direction and magnitude of the pooled effect remained stable across all nine outcomes. For the 3000m run, excluding Yan, 2024 reduced the SMD from −0.87 to −0.52 [−0.68, −0.36] while remaining statistically significant. For FMS, excluding Yan, 2024 reduced I^2^ from 93.2% to 0%, confirming that the training approach (comprehensive vs. FMS-specific) was the key source of heterogeneity. Excluding the two non-randomized studies (Wu et al., 2021 and Zhang et al., 2011) for the injury outcome yielded RR = 0.65 [0.46, 0.91], consistent with the primary analysis.

### 3.7 Publication bias

Egger’s test was non-significant for pull-ups (p = 0.182, 19 studies), but the funnel plot showed slight asymmetry; therefore, we performed a Trim-and-Fill analysis, which yielded an adjusted SMD of 0.40 [0.22, 0.58], still indicating a significant effect. Egger’s test was also non-significant for the 100m sprint (p > 0.05, 10 studies). For the 3000m run (15 studies), Egger’s test was non-significant (p = 0.10), suggesting no strong evidence of publication bias. Trim-and-fill adjustment for the 3000m run estimated an adjusted SMD of approximately −0.60, consistent in direction with the primary analysis. The remaining outcomes (with <10 studies) showed broadly symmetrical funnel plots, and trim-and-fill corrections did not alter the direction or significance of the pooled effects. Overall, publication bias was judged to be low.

## 4 Discussion

### 4.1 Summary of main findings

This is the first systematic review and meta-analysis conducted specifically for the Chinese People’s Armed Police Force (PAP), rigorously following the PICOS framework and prospective registration (Page et al., 2021). The key findings are twofold: (1) Modern physical training significantly outperformed conventional training across all eight physical fitness outcomes, with effect sizes ranging from SMD = −0.42 (100m sprint) to SMD = 1.92 (FMS total score). The latter, together with parallel bar dips (SMD = 0.58), reached large effect magnitudes. (2) Training-related injury risk was reduced by 37% (RR = 0.63 [0.46, 0.87], I^2^ = 9.9%). This dual benefit—simultaneously improving performance and reducing injuries—represents a distinct advantage of modern physical training over conventional approaches. These findings are consistent with the military training meta-analysis by Smith et al. (2022) in terms of effect size range.

### 4.2 Comparison with previous studies

The direction of our results aligns with the preliminary findings of three similar PROSPERO registrations (CRD42025636689, CRD420261442021, CRD420261415027). However, this study possesses three distinctive features: first, population specificity—while the three registrations cover mixed multinational military or tactical populations, our study focuses exclusively on the Chinese PAP, enhancing applicability within this force; second, dual-outcome coverage—our study simultaneously examines physical performance and injury rates, providing an integrated effectiveness-and-safety evaluation; third, refined subgroup analysis—beyond the conventional population and duration dimensions, we further stratified FMS outcomes by training approach (specific vs. comprehensive) and injury outcomes by study design (RCT vs. non-randomized). At the effect size level, our pull-up result (SMD = 0.68) is comparable to a multinational military meta-analysis (SMD ≈ 0.5–0.7) (Smith et al., 2022), while our 3000m run effect (SMD = −0.87) exceeds comparable estimates (Friedl et al., 2015), potentially reflecting lower baseline aerobic fitness in PAP personnel and the high intensity of local training protocols. In interpreting these results, it should be noted that significant publication bias was not detected for pull-ups (Egger’s p = 0.182), although the funnel plot showed slight asymmetry. A subsequent trim-and-fill analysis yielded an adjusted SMD of 0.40 [0.22, 0.58], still indicating a significant effect. Caution is warranted when interpreting the magnitude of the pull-up effect, and readers are referred to the funnel plot in **Supplementary Figure 5** for visual assessment. A recent meta-analysis of operational military training also reported positive adaptations in physical performance (Aandstad et al., 2020; Murray et al., 2025), consistent with our findings.

### 4.3 Sources of heterogeneity and training implications

The high heterogeneity of the 3000m run (I^2^ = 94%) was primarily attributable to the extreme effect of Yan (2024) (Yan, 2024) (ECP, SMD = −4.12). Even after exclusion, heterogeneity remained at 50.6%, indicating that additional moderators beyond this single outlier contributed to between-study variation. A key physiological factor is differential training responsiveness mediated by baseline aerobic capacity: individuals with lower initial VO_2_max tend to exhibit greater relative improvements (ceiling effect) (Smith et al., 2022), and the 20 included studies encompassed populations ranging from newly enlisted recruits with limited aerobic conditioning to veteran special forces personnel with established fitness profiles. Furthermore, PAP units are stationed across diverse geographic regions of China, with elevations spanning from sea level to the Qinghai–Tibet Plateau (>3,000 m) (Zhang et al., 2017); altitude-related differences in oxygen availability directly affect both absolute 3000m run times and the magnitude of training-induced aerobic adaptations. Regional variation in training culture, testing protocols (e.g., track surface, pacing guidance, motivational encouragement during maximal-effort testing), and the consistency of test administration further amplify between-study variability. These sources of heterogeneity are consistent with the well-documented inter-individual variation in physiological adaptations during military training (Ojanen et al., 2020) and the modulating role of individual training load (Jurvelin et al., 2019).

The FMS heterogeneity (I^2^ = 93%) carries a clear training implication. It was entirely explained by the difference between comprehensive training (ECP programs that improve FMS as a byproduct, SMD = 0.90) and FMS-specific corrective programs that target identified movement deficiencies, SMD ≈ 2.36; because corrective exercise is prescribed to the specific movement patterns that scored lowest at screening, the observed gains are concentrated in those deficient sub-scores rather than uniformly distributed across all seven components. Restricting the analysis to FMS-specific programs eliminated heterogeneity entirely (I^2^ = 0%). Furthermore, <12-week FMS-specific programs produced a substantially larger effect (SMD = 2.45) than ≥12-week comprehensive programs (SMD = 0.90, p < 0.001). Basar et al. (2018) similarly reported significant FMS improvements following corrective exercise interventions in Reserve Officers’ Training Corps (ROTC) cadets. Alternative training modalities, such as suspension training, have also shown promise for improving movement quality in military personnel (Mozafaripour et al., 2024). These findings indicate that the value of FMS screening lies not in the screening itself, but in the subsequent prescription of targeted corrective exercises—a principle with direct applicability to the recruit training setting where injury rates are highest.

### 4.4 Tiered training recommendations

Based on our subgroup analyses, we propose the following tiered recommendations: (1) During recruit training (<12 weeks), priority should be given to FMS screening followed by targeted corrective exercise programs, given their large effect on FMS improvement (SMD = 2.45) and concurrent injury risk reduction of 35–37%. This is consistent with the preventive training approach validated in tactical athlete programs (Sell et al., 2016; Vähi et al., 2023). (2) For ongoing training in veterans and special forces personnel (≥12 weeks), periodized CPT programs (ECP, periodized strength, lactate-threshold training) are recommended, as this population showed larger effects on pull-ups (SMD = 0.81) and the 3000m run (SMD = −1.00) than recruits. Concurrent and periodized training models have also demonstrated efficacy in British Army (Nevin et al., 2026), combined training interventions (Pihlainen et al., 2020), and general military (Petré et al., 2021) populations; the distribution of training sessions may further optimize these adaptations (Kilen et al., 2021). Where resources permit, periodic FMS screening could also be considered for veterans, as movement-quality deficits and compensatory patterns may persist or emerge after initial training; however, the available evidence does not yet allow us to quantify the benefits of FMS-guided correction in this population. (3) A comprehensive, training-cycle-wide preventive mechanism incorporating medical supervision, corrective training, and health education should be established, as our data and prior evidence (Wang et al., 2022; Thornton et al., 2024) suggest that PT interventions can reduce injury rates by approximately 35%. To facilitate implementation in resource-constrained settings, minimal-equipment resistance training protocols may serve as a practical alternative (Cintineo et al., 2023). Finally, objective physiological monitoring tools—such as heart rate variability (HRV) assessment—warrant investigation in future research to guide individualized training intensity (Tomes et al., 2020). In military settings, HRV has been proposed as a practical, non-invasive index of autonomic recovery for monitoring training load and detecting early signs of overtraining, which may help prevent injuries and performance decrements during intensive conditioning periods.

### 4.5 Strengths and limitations

Key strengths of this study include: (1) the first PAP-specific systematic review with prospective PROSPERO registration and rigorous PRISMA/GRADE methodology; (2) a dual-outcome (performance + injury) design, providing a more comprehensive evaluation than single-domain meta-analyses (Smith et al., 2022; Lan and Shu, 2025); (3) complete sensitivity analyses (leave-one-out, trim-and-fill, Egger’s test); and (4) refined subgroup analyses that identify training-approach-specific effects.

Limitations include: (1) several outcomes had few contributing studies (FMS k = 4, training injury k = 4, parallel bar dips k = 7; the injury prevention effect showed a consistent trend in RCTs only (RR = 0.69) but with wide confidence intervals (k=2), zigzag run k = 6), limiting statistical power for subgroup analyses; (2) two non-randomized studies (Zhang et al., 2011; Wu et al., 2021) were assessed as having “serious” or “moderate” risk of bias (ROBINS-I), reducing confidence in the injury outcome; (3) all participants were male, precluding generalization to female PAP personnel; (4) the primary studies provided limited quantitative detail on the conventional training programs (**Supplementary Table 4** in the Supplementary Materials): only one study reported training frequency and volume, and none reported training intensity, preventing dose-based comparisons between conventional and modern training; (5) substantial heterogeneity in training programs precluded precise dose–response quantification; (6) Egger’s test for the 3000m run was non-significant (p = 0.10), suggesting no strong evidence of publication bias; and (7) exercise training studies cannot be blinded, introducing potential performance and measurement bias; and (8) of the 20 included studies, 12 (60%) were master’s theses, whose methodological details—particularly allocation concealment and outcome assessment blinding—could not always be fully verified from the available reports, although all studies underwent formal risk-of-bias assessment and the pooled results were robust to leave-one-out sensitivity analyses; most of these theses are indexed academic databases (e.g., CNKI and Wanfang) but have not undergone formal international peer review, and we therefore encourage cautious interpretation of these studies and recommend that future syntheses prioritize journal-published evidence where possible; and (9) the included studies did not separate injury counts by training mode, so it was not possible to determine whether training-related injuries are more frequent during physical conditioning sessions or during technical/drill routines; future studies should disaggregate injury data by training component to address this question.

## 5 Conclusions

This systematic review and meta-analysis demonstrates that modern physical training (CPT and PT) significantly enhances physical performance across multiple dimensions—including upper body strength, upper body pushing power, core endurance, aerobic capacity, speed, agility, functional movement and body composition—and reduces the risk of training-related injuries by approximately 37% People’s Armed Police Force personnel (RR = 0.63, 95% CI [0.46, 0.87]). These effects are consistent across recruits and veterans and across short (<12 weeks) and longer (≥12 weeks) intervention durations. The finding that FMS-specific corrective training yields substantially larger effects than comprehensive programs (subgroup difference p < 0.001) has direct implications for recruit training. We recommend that PAP training programs incorporate FMS-guided corrective exercise during initial training and adopt periodized comprehensive training during ongoing training, supported by a training-cycle-wide preventive mechanism to achieve the dual goals of enhanced performance and reduced training-related injury risk.

## Data Availability

The original contributions presented in the study are included in the article/**Supplementary Material**. Further inquiries can be directed to the corresponding author.
