# Supplementary material for: Effects of modern physical training on physical performance and training-related injuries in the Chinese people’s armed police force: a systematic review and meta-analysis
Source: Front Physiol. 2026 Sep 14;17:1953076. doi: 10.3389/fphys.2026.1953076 (PMC13616791; doi:10.3389/fphys.2026.1953076)
Supplement: Supplementary file 1 [file SupplementaryFile1.docx]

**Supplementary Materials**

*Effects of Modern Physical Training on Physical Performance and Training-Related Injuries
in the Chinese People’s Armed Police Force: A Systematic Review and Meta-Analysis*

PROSPERO: CRD420261448648

This supplementary materials file contains additional data supporting the findings of the main manuscript, including detailed study characteristics, GRADE evidence profile, forest plots for all secondary outcomes, subgroup analyses, sensitivity analyses, and publication bias assessments.

**A. Supplementary Tables**

**Table S1. Detailed characteristics of the 20 included studies**

*CAP, Chinese People's Armed Police Force (PAP); CPT, Comprehensive Physical Training; PT, Preventive Training; RCT, Randomized Controlled Trial; EG, Experimental Group; CG, Control Group; FMS, Functional Movement Screen.*

| **Study** | **Training Type** | **Population** | **Duration (weeks)** | **Study Design** | **Sample Size (n, EG/CG)** | **Intervention** | **Control** | **Outcomes Measured** |
| --- | --- | --- | --- | --- | --- | --- | --- | --- |
| **Yan (2024)** | CPT | Recruits | ≥12 | RCT | 200 (100/100) | 15-week CrossFit training | Conventional physical training | Body weight, BMI, FMS (7 items), 3000m run, pull-ups, parallel bar dips, sit-ups, basic fitness combo |
| **Li (2022)** | CPT | Veterans | ≥12 | RCT | 120 (60/60) | Periodized pattern training (periodized physical fitness) | Traditional physical fitness training | 100m sprint, weighted squat, pull-ups, 3000m run, forward bend |
| **Chen (2020)** | CPT | Recruits | <12 | RCT | 44 (22/22) | 10-week new physical training (strength + endurance + speed + agility) | 10-week routine military physical training | Push-ups, pull-ups, 100m sprint, 3000m run, shuttle run |
| **Zhou (2018)** | CPT | Recruits | ≥12 | RCT | 61 (31/30) | Basic physical training (strength + endurance + speed + flexibility) | Routine physical training | Zigzag run, pull-ups, 3000m run, sit-ups |
| **Guo (2020)** | CPT | Recruits | <12 | RCT | 140 (70/70) | 8-week weekly-cycle optimized military physical training (6 sessions/wk, 100min/session) | Original military physical training syllabus | Physiological indicators, basic fitness battery, military fitness assessment |
| **Wu (2021)** | PT | Recruits | <12 | Non-RCT (cluster) | 316 (158/158) | EMPF screening + individualized corrective exercise program on top of 8-week routine training | 8-week routine military training only | Training injury rate, physical fitness indicators |
| **Zhang (2005a)** | CPT | Recruits | <12 | RCT | 400 (200/200) | 8-week intervention: sustained run (3-5km) + interval run (400-600m) alternating; circuit strength training (8-10 exercises); speed & flexibility | Current military training program (5km sustained run + single-exercise strength) | Pull-ups, sit-ups (3min), 1-leg squat, back strength, grip strength, standing long jump, 100m sprint, 3000m run, cross jump, trunk forward flexion, VO2peak |
| **Zhang (2005b)** | CPT | Veterans | <12 | RCT | 210 (120/90) | Same 8-week intervention as Zhang (2005a) | Current military training program | Same as Zhang (2005a) |
| **Zhang (2005c)** | CPT | Cadets | <12 | RCT | 380 (200/180) | Same 8-week intervention as Zhang (2005a) | Routine training program | Same as Zhang (2005a) + 1500m run |
| **Zhang (2022)** | PT | Recruits | <12 | RCT | 60 (30/30) | 8-week functional fitness training (5 sessions/wk, 60min/session; basic movement + general fitness + exam-oriented stages) | 8-week traditional physical training (original military plan: calisthenics, barbells, long-distance running) | FMS total score, pull-ups, parallel bar dips, basic fitness combo 1&2, 3000m run, training injury |
| **Wang (2021)** | PT | Recruits | ≥12 | RCT | 150 (75/75) | 12-week functional training (6 sessions/wk, 2hr/session; strength, core, anaerobic/aerobic, agility, flexibility, movement pattern) | 12-week traditional physical training | BMI, 3000m run (min), 30m×2 zigzag run (s), pull-ups, sit-ups (2min), FMS total and sub-scores, physical fitness standards |
| **Li (2021)** | CPT | Veterans | <12 | RCT | 50 (26/24) | 8-week modern physical training (strength, speed, core, FMS corrective exercises, 5 sessions/wk) | 8-week traditional military physical training | Pull-ups, sit-ups, 30m×2 zigzag run, 3000m run, 400m obstacle run, FMS total score |
| **Zhang (2017)** | CPT | Veterans | <12 | RCT | 40 (20/20) | 4-week adaptive physical training (optimized morning + afternoon sessions; hypoxic long-distance, rapid response, weighted marching) | 4-week routine physical training | 5km armed越野 (s), 800m run (s), 400m obstacle (s), pull-ups, push-ups |
| **Hao (2005)** | PT | Recruits | ≥12 | Stratified RCT | 835 (420/415) | 3-month health promotion program (scientific training structure, 3-phase periodization, recovery methods, psychological counseling, leadership supervision) | 3-month routine military physical training | Training injury rate, back strength, flexed arm hang, 3000m run |
| **Hu (2021)** | CPT | Veterans | <12 | RCT | 60 (30/30) | 10-week new physical training (special preparation, stratified training, regeneration & recovery) | 10-week original training plan | Pull-ups, 100m sprint, 5km armed越野 (s) |
| **Duan (2014)** | CPT | Recruits | ≥12 | RCT | 56 (28/28) | 12-week lactate threshold-monitored aerobic endurance + circuit strength training | 12-week traditional experience-based training | 3000m run (s), pull-ups, parallel bar dips, 5km armed越野 (s) |
| **Liu (2017)** | PT | Veterans | <12 | Cluster RCT | 41 (20/21) | 10-week core strength and stability-focused training | 10-week traditional physical training | Push-ups, sit-ups, 5000m weighted run (s) |
| **Chen (2022)** | CPT | Recruits | ≥12 | RCT | 150 (75/75) | 12-week modern physical training (strength, speed, endurance, agility, flexibility, coordination + FMS corrective) | 12-week original training plan | BMI, 3000m run (s), 30m×2 zigzag run (s), pull-ups, sit-ups (2min) |
| **Cao (2021)** | CPT | Recruits | ≥12 | RCT | 16 (8/8) | 12-week lactate threshold training (4 sessions/wk, 40min/session, HR-monitored, combined strength) | 12-week routine special police training | 3km run (s), BMI, pull-ups, sit-ups |
| **Zhang (2011)** | PT | Veterans | <12 (6mo) | Non-RCT (cluster) | 943 (429/514) | 6-month comprehensive medical intervention (health education, on-site medical supervision, warm-up/cool-down standardization, recovery guidance, psychological intervention) | Routine training mode | Training injury rate (event counts) |

Note: "s" = seconds. Zhang (2005a), (2005b), and (2005c) are three independent cohorts from the same investigator. Duration ≥12 weeks includes studies of 3 months (Hao 2005) and 6 months (Zhang 2011).

**Table S2. GRADE Evidence Profile**

*Modern physical training versus conventional military training for the Chinese People’s Armed Police Force (PAP)*

| **Outcome (No. of studies)** | **Study design(s)** | **Risk of bias** | **Inconsistency (I²)** | **Indirectness** | **Imprecision** | **Publication bias** | **Overall certainty** | **Effect size (95% CI)** |
| --- | --- | --- | --- | --- | --- | --- | --- | --- |
| **Pull-ups (19)** | RCT | Some concerns (not serious−1) | I² = 76.5% (moderate−1) | Not serious (0) | Not serious (0) | Not detected (0) | Low (downgraded 2) | SMD = 0.68 [0.50, 0.85] |
| **Parallel bar dips (7)** | RCT | Some concerns (not serious−1) | I² = 74.9% (moderate−1) | Not serious (0) | Not serious (0) | Undetected (0) | Low (downgraded 2) | SMD = 0.58 [0.16, 0.99] |
| **2-min sit-ups (9)** | RCT | Some concerns (not serious−1) | I² = 81.1% (substantial−1) | Not serious (0) | Not serious (0) | Undetected (0) | Low (downgraded 2) | SMD = 0.57 [0.24, 0.89] |
| **3000m run (15)** | RCT | Some concerns (not serious−1) | I² = 94.0% (very high−2) | Not serious (0) | Not serious (0) | Suspected (not serious−1) | Very low (downgraded 4) | SMD = −0.87 [−1.40, −0.33] |
| **100m sprint (10)** | RCT | Some concerns (not serious−1) | I² = 63.3% (moderate−1) | Not serious (0) | Not serious (0) | Not detected (0) | Low (downgraded 2) | SMD = −0.42 [−0.65, −0.19] |
| **30m×2 zigzag run (6)** | RCT | Some concerns (not serious−1) | I² = 45.6% (not serious−0) | Not serious (0) | Serious (small k)−1 | Undetected (0) | Low (downgraded 2) | SMD = −0.82 [−1.16, −0.48] |
| **FMS total score (4)** | RCT | Some concerns (not serious−1) | I² = 93.2% (very high−2) | Not serious (0) | Serious (1 outlier)−1 | Undetected (0) | Very low (downgraded 4) | SMD = 1.92 [0.68, 3.17] |
| **BMI (5)** | RCT | Some concerns (not serious−1) | I² = 0% (not serious−0) | Not serious (0) | Serious (small ES)−1 | Undetected (0) | Low (downgraded 2) | SMD = 0.19 [0.03, 0.36] |
| **Training- related injury rate (4)** | RCT + NRS | Serious (NRS included−2) | I² = 9.9% (not serious−0) | Not serious (0) | Not serious (0) | Undetected (0) | Very low (downgraded 2) | RR = 0.63 [0.46, 0.87] |

Note. The numbers in parentheses after each inconsistency or imprecision rating indicate the number of levels downgraded. The overall certainty is determined by the total number of downgrades: 0 = High, 1–2 = Moderate, 3–4 = Low, >4 = Very Low. "Not serious" (0) = no downgrade; "Serious" (−1) = one level downgrade; "Very serious" (−2) = two levels downgrade. Abbreviations: RCT = randomized controlled trial; NRS = non-randomized study; SMD = standardized mean difference; RR = risk ratio; CI = confidence interval; k = number of studies; ES = effect size. Small k (k < 10) and 1 outlier refer to limitations that reduce confidence in the precision of the estimate. Small ES = the effect size is smaller than the minimal clinically important difference.

**Table S3. Egger’s Regression Test for Publication Bias
(Outcomes with ≥10 Included Studies)**

| **Outcome** | **k** | **t** | **df** | **P-value** | **Interpretation** |
| --- | --- | --- | --- | --- | --- |
| **Pull-ups (Upper body strength)** | 19 | — | 16 | 0.182 | Non-significant. Funnel plot showed slight asymmetry; Trim-and-Fill analysis yielded an adjusted SMD of 0.40 [0.22, 0.58], which remained statistically significant. |
| **3000 m run (Aerobic endurance)** | 15 | −1.76 | 13 | 0.1025 | Non-significant. No strong evidence of publication bias. The high residual heterogeneity (τ² = 14.53) suggests between-study variation rather than selective reporting as the dominant source of funnel asymmetry. |
| **100 m sprint (Speed)** | 10 | — | 8 | > 0.05 | Non-significant. Symmetrical funnel plot (visual inspection). |
| **Summary** | 19–10 | — | 16–8 | All > 0.05 | All three outcomes with ≥10 included studies showed no statistically significant Egger’s test results, indicating no strong evidence of publication bias in this review. |

Note. Egger’s regression test examines funnel plot asymmetry by regressing the standard normal deviate (SND = effect / SE) against the standard error (SE), weighted by inverse variance. A non-significant P-value (P ≥ 0.05) indicates no statistically detectable asymmetry. For pull-ups, although the Egger test was non-significant, the funnel plot showed mild asymmetry, and a subsequent Trim-and-Fill analysis was performed as a sensitivity analysis. Only outcomes with k ≥ 10 studies were tested, as recommended in the Cochrane Handbook (Page et al., 2021), because tests based on fewer than 10 studies lack sufficient statistical power to detect asymmetry.

**Table S4. Characteristics of the Conventional (Control) Physical Training Programs in the 20 Included Studies**

| **Study** | **Population** | **Conventional program content** | **Frequency** | **Volume** | **Intensity** | **Periodization/Progression** | **Duration** |
| --- | --- | --- | --- | --- | --- | --- | --- |
| Yan (2024) | Recruits | Conventional basic fitness training (long-distance running, push-ups, pull-ups, drill routines) | NR | NR | NR | NR | 15 weeks |
| Zhang (2005a) | Recruits | Existing military training plan (conventional regimen) | NR | NR | NR | NR | 8 weeks |
| Zhang (2005b) | Recruits & veterans | Existing training plan (conventional regimen) | 5 sessions/week | Preparation 15 min + training 40 min | NR | NR | 8 weeks |
| Zhang (2005c) | Cadets | Conventional training plan | NR | NR | NR | NR | NR |
| Zhang (2022) | Recruits | Original unit traditional physical training plan | NR | NR | NR | NR | 8 weeks |
| Wang (2021) | Recruits | Routine physical training content of the mobile PAP unit | NR | NR | NR | NR | NR |
| Wu (2021) | Recruits | Conventional recruit physical training | NR | NR | NR | NR | NR |
| Guo (2020) | Recruits | Original military fitness training plan | NR | NR | NR | Three-stage design | NR |
| Li (2021) | Veterans | Current traditional military physical training plan | NR | NR | NR | NR | NR |
| Li (2022) | Veterans | Traditional high-volume training (conventional training method) | NR | NR | NR | NR | 16 weeks |
| Zhang (2017) | Veterans | Conventional training at high-altitude duty station | NR | NR | NR | NR | NR |
| Cao (2021) | Recruits | Original special police unit training tasks | NR | NR | NR | NR | 12 weeks |
| Hao (2005) | Recruits | Conventional military training requirements of the unit | NR | NR | NR | NR | Basic training period |
| Zhou (2018) | Recruits | Traditional basic military physical training | NR | NR | NR | NR | NR |
| Hu (2021) | Veterans | Conventional unit physical training plan (traditional three-stage model) | NR | NR | NR | Traditional three-stage model | 10 weeks |
| Chen (2020) | Recruits | Original unit physical training plan | NR | NR | NR | NR | 10 weeks |
| Duan (2014) | Recruits | Traditional experience-based training method | NR | NR | NR | NR | 12 weeks |
| Liu (2017) | Veterans (firefighters) | Conventional training means (similar number of methods as intervention) | NR | NR | NR | NR | 10 weeks |
| Chen (2022) | Recruits | Original training plan executed by the same instructors | NR | NR | NR | NR | 12 weeks |
| Zhang (2011) | Veterans | Conventional training methods without additional medical intervention | NR | NR | NR | NR | Basic training cycle |
| **Summary** | **20 studies** | **All 20 studies used conventional military training as control; most described it only in general terms** | **Reported in 1/20 (5%)** | **Reported in 1/20 (5%)** | **Reported in 0/20 (0%)** | **Reported in 2/20 (10%)** | **Reported in 13/20 (65%)** |

Note. NR = not reported in the original study. Only one study (5%) reported training frequency (Zhang 2005b), only one study (5%) reported training volume, no study reported training intensity, and only two studies (10%) reported the periodization/progression model. This limited reporting of conventional training characteristics in the primary studies prevented quantitative synthesis of training dose, which we acknowledge as an important limitation of the available evidence.

**B. Supplementary Figures**

**B.1. Forest Plots of Pull-ups**

Figure S1. Subgroup analysis by population for pull-ups (19 studies)

Figure S2. Subgroup analysis by intervention duration for pull-ups (19 studies)

Figure S3. Leave-one-out sensitivity analysis for pull-ups (19 studies)

Figure S4. Funnel plot for pull-ups (19 studies)

Figure S5. Trim-and-fill funnel plot for pull-ups (19 studies)

**B.2. Forest Plots of Parallel bar dips**

Figure S6. Forest plot for parallel bar dips (7 studies)

Figure S7. Subgroup analysis by population for parallel bar dips (7 studies)

Figure S8. Subgroup analysis by intervention duration for parallel bar dips (7 studies)

Figure S9. Leave-one-out sensitivity analysis for parallel bar dips (7 studies)

Figure S10. Funnel plot for parallel bar dips (7 studies)

Figure S11. Trim-and-fill funnel plot for parallel bar dips (7 studies)

**B.3. Forest Plots of 2-minute sit-ups**

Figure S12. Forest plot for 2-minute sit-ups (9 studies)

Figure S13. Subgroup analysis by population for 2-minute sit-ups (9 studies)

Figure S14. Subgroup analysis by intervention duration for 2-minute sit-ups (9 studies)

Figure S15. Leave-one-out sensitivity analysis for 2-minute sit-ups (9 studies)

Figure S16. Funnel plot for 2-minute sit-ups (9 studies)

Figure S17. Trim-and-fill funnel plot for 2-minute sit-ups (9 studies)

**B.4. Forest Plots of 3000m run**

Figure S18. Forest plot for 3000 m run (15 studies)

Figure S19. Subgroup analysis by population for 3000 m run (15 studies)

Figure S20. Subgroup analysis by intervention duration for 3000 m run (15 studies)

Figure S21. Leave-one-out sensitivity analysis for 3000 m run (15 studies)

Figure S22. Funnel plot for 3000 m run (15 studies)

Figure S23. Trim-and-fill funnel plot for 3000 m run (15 studies)

**B.5. Forest Plots of 3000 m Run (Excluding Yan 2024)**

Figure S24. Forest plot for 3000 m run (excluding Yan 2024, 14 studies)

Figure S25. Subgroup analysis by population for 3000 m run (excluding Yan 2024)

Figure S26. Subgroup analysis by intervention duration for 3000 m run (excluding Yan 2024)

Figure S27. Leave-one-out sensitivity analysis for 3000 m run (excluding Yan 2024)

Figure S28. Funnel plot for 3000 m run (excluding Yan 2024)

Figure S29. Trim-and-fill funnel plot for 3000 m run (excluding Yan 2024)

**B.6. Forest Plots of 100m sprint**

Figure S30. Forest plot for 100 m sprint (10 studies)

Figure S31. Subgroup analysis by population for 100 m sprint (10 studies)

Figure S32. Subgroup analysis by intervention duration for 100 m sprint (10 studies)

Figure S33. Leave-one-out sensitivity analysis for 100 m sprint (10 studies)

Figure S34. Funnel plot for 100 m sprint (10 studies)

Figure S35. Trim-and-fill funnel plot for 100 m sprint (10 studies)

**B.7. Forest Plots of 30m × 2 zigzag run**

Figure S36. Forest plot for 30 m × 2 zigzag run (6 studies)

Figure S37. Subgroup analysis by population for 30 m × 2 zigzag run (6 studies)

Figure S38. Subgroup analysis by intervention duration for 30 m × 2 zigzag run (6 studies)

Figure S39. Leave-one-out sensitivity analysis for 30 m × 2 zigzag run (6 studies)

Figure S40. Funnel plot for 30 m × 2 zigzag run (6 studies)

Figure S41. Trim-and-fill funnel plot for 30 m × 2 zigzag run (6 studies)

**B.8. Forest Plots of FMS total score**

Figure S42. Forest plot for FMS total score (4 studies)

Figure S43. Subgroup analysis by intervention duration for FMS total score (4 studies)

Figure S44. Leave-one-out sensitivity analysis for FMS total score (4 studies)

Figure S45. Funnel plot for FMS total score (4 studies)

Figure S46. Trim-and-fill funnel plot for FMS total score (4 studies)

**B.9. Forest Plots of BMI**

Figure S47. Forest plot for BMI (5 studies)

Figure S48. Leave-one-out sensitivity analysis for BMI (5 studies)

Figure S49. Funnel plot for BMI (5 studies)

Figure S50. Trim-and-fill funnel plot for BMI (5 studies)

**B.10. Forest Plots of Training-related injury rate**

Figure S51. Forest plot for training-related injury rate (4 studies)

Figure S52. Subgroup analysis by population for training-related injury rate (4 studies)

Figure S53. Subgroup analysis by intervention duration for training-related injury rate (4 studies)

Figure S54. Subgroup analysis by study design for training-related injury rate (RCT vs. NRS)

Figure S55. Leave-one-out sensitivity analysis for training-related injury rate (4 studies)

Figure S56. Funnel plot for training-related injury rate (4 studies)

Figure S57. Trim-and-fill funnel plot for training-related injury rate (4 studies)
